# Moderator role of self-compassion in the relationship between borderline features and suicide ideation in adolescents

**DOI:** 10.1192/j.eurpsy.2021.585

**Published:** 2021-08-13

**Authors:** C. Pinto-Gouveia, D. Carreiras, A. Rocheteaux, A. Valente, P. Castilho, M. Cunha

**Affiliations:** 1 Psychiatry Department, Centro Hospitalar e Universitário de Coimbra, Coimbra, Portugal; 2 Center For Research In Neuropsychology And Cognitive And Behavioral Intervention, University of Coimbra, Coimbra, Portugal; 3 Clinical Psychology Department, Miguel Torga Institute of Higher Education, Coimbra, Portugal

**Keywords:** Self-compassion, Suicide ideation, Borderline features

## Abstract

**Introduction:**

Borderline Personality Disorder (BPD) is an impairing disorder with distinct features such as instability in self-image, relationships and affect. Considering the developmental nature of BPD, borderline features are not rarely exhibited in adolescence. These features tend to be associated with depression and suicide ideation, as well as with a negative self-to-self relationship. Self-compassion has been consistently identified as a positive attitude with oneself when experiencing suffering.

**Objectives:**

The aim of the current study was to explore the role of self-compassion in the relationship between borderline features and suicide ideation, when controlled depressive symptoms.

**Methods:**

Sample was composed by 665 adolescents (58.5% females and 41.5% males), with a mean of 15.54 years of age (SD = 1.52), who completed self-report questionnaires. Data was analyzed using SPSS (version 23) and PROCESS Macro.

**Results:**

showed that girls presented higher borderline features and suicide ideation and lower self-compassion compared to boys. The moderation model explained 66% of suicide ideation, with gender and depression as covariates. The interaction of borderline features and self-compassion had a unique and significant effect on suicide ideation, when controlled depression and gender. Adolescents with higher levels of borderline features and lower self-compassion presented significantly higher suicide ideation, compared to those with higher self-compassion.

**Conclusions:**

These findings suggest that developing self-compassion in adolescents with evident borderline features might attenuate their tendency to think about committing suicide.

